# First-principles investigation on the segregation of Pd at LaFe_1-*x*_Pd_*x*_O_3-y_ surfaces

**DOI:** 10.1186/1556-276X-8-203

**Published:** 2013-05-01

**Authors:** Zhi-xue Tian, Akifumi Uozumi, Ikutaro Hamada, Susumu Yanagisawa, Hidetoshi Kizaki, Kouji Inagaki, Yoshitada Morikawa

**Affiliations:** 1 Division of Precision Science & Technology and Applied Physics, Graduate School of Engineering, Osaka University, 2-1, Yamada-okaSuita, Osaka 565-0871, Japan; 2 Insitute of Scientific and Industrial Research, Osaka University, 8-1 MihogaokaIbaraki, Osaka 567-0047, Japan; 3 Advanced Institute for Materials Research (AIMR), Tohoku University, Sendai 980-8577, Japan; 4 Department of Physics and Earth Science, Faculty of Science, University of the Ryukyus, 1 SenbaruNishihara, Okinawa 903-0213, Japan; 5 Elements Strategy Initiative for Catalysts and Batteries (ESICB), Kyoto University, Katsura, Kyoto 615-8520, Japan

**Keywords:** Perovskite, LaFeO_3_, Palladium, Density functional theory, Surface segregation

## Abstract

First-principles calculations were performed to investigate the effect of Pd concentration and oxygen vacancies on the stability of Pd at LaFeO_3_ surfaces. We found a much stronger tendency of Pd to segregate by taking the aggregation of Pd at LaFe_1-*x*_Pd_*x*_O_3-*y*_ surfaces into consideration, resulting in a pair of Pd-Pd around a vacancy. Moreover, we predicted that one oxygen-vacancy-containing FeO_2_-terminated surfaces would be stable at high temperatures by comparing the stability of LaFe_1-*x*_Pd_*x*_O_3-*y*_ surfaces, which further supports our previous conclusion that a Pd-containing perovskite catalyst should be calcined at 1,073 K or higher temperatures in air to enhance the segregation of Pd in the vicinity of surfaces to rapidly transform the Pd catalyst from oxidized to reduced states on the perovskite support.

## Background

A three-way catalyst simultaneously transforms toxic exhaust emissions from motor vehicles into harmless gases. However, the sintering problem, i.e., the growth and agglomeration of precious metal particles on conventional catalysts during vehicle use dramatically degrades catalytic activity, and large amounts of precious metals are required to retain the activity of catalysts after long periods of use. Thus, intelligent catalysts have attracted worldwide attention due to their greatly improved durability as a result of the self-regenerative function of precious metal nanoparticles [1-3]. It has been confirmed that the activity of catalysts can be preserved, and the amount of precious metals that are required can be reduced by 70% to 90% [4,5]. The self-regenerative function, which can be explained as resulting from the transformation of the state of precious metals (Pd, Pt, and Rh) that reversibly move into and out of the LaFe_1*-x*_M_*x*_O_3_ perovskite lattice, significantly suppresses the growth of precious metals during the use of catalysts.

Thus far, many experiments have been devoted to research on the state of Pd in perovskite in redox processes. Uenishi et al. [6] investigated the superior start-up activity of LaFePdO_*x*_ at low temperatures (from 100°C to 400°C) using X-ray spectroscopic techniques under the practical conditions where they controlled automotive emissions. They found the Pd^0^ phase partially segregated outside the surface even at low temperatures; thus, the segregation of Pd^0^ under a reductive atmosphere induced the start-up activity of LaFePdO_*x*_. Eyssler et al. found a high concentration of Pd distributed on the LaFeO_3_ (LFO) surface that contributed to high methane combustion due to the formation of PdO in which Pd^2+^ was in square planar coordination. Additionally, two Pd species (Pd^2+^ at the surface and Pd^3+^ in a solid solution) were found to be generated in further calcination. Pd^2+^ and Pd^3+^ could be transformed in equilibrium under thermal treatment conditions [7,8]. More recently, Eyssler et al. studied the state of Pd in different B-site substitutions and compared the effect of catalytic activities on methane combustion. A well-dispersed octahedral Pd-O species was found for Fe- and Co B- site cations, and PdO particles were on the LaMnO_3_ surface [9]. Above all, related investigations have become more important as the activity of catalysts strongly depends on the state of the precipitated Pd.

Hamada et al. [10] more recently found in a density functional theory (DFT) investigation that oxygen vacancies (V_O_s) that formed near the LFO surface promoted the segregation of Pd. They also proposed a scenario that perovskite containing precious metal is calcined during the catalyst preparation step at 1,073 K for 2 h in air, and then V_O_s are produced that enhance Pd segregation, resulting in a LaPdO_3-*y*_ layer that eventually forms close to the surface. The LaPdO_3-*y*_ layer in the vicinity of the surface promotes efficient switching between Pd metal particles under reductive conditions and the dissolved state of Pd in the LaFe_1-*x*_Pd_*x*_O_3_ perovskite lattice under oxidative conditions. Therefore, the LaPdO_3-*y*_ layers formed in the vicinity of the oxide surface play a key role in the self-regenerative function. Almost simultaneously, transmission electron microscopy observations [11] of atomic-scale processes in Pd-LFO catalysts have demonstrated that redox reactions between the formation of Pd particles on the Pd-LFO surface under reducing conditions and the dissolution of Pd particles into LFO under oxidizing conditions take place in spatially-limited areas, especially in the proximity of oxide surfaces, indicating strong interactions between Pd and oxide surfaces. Katz's results also provided strong support for the mechanism proposed by Hamada et al. However, the stability of the LaPdO_3-*y*_ layer and the mechanism for Pd leaving the LaPdO_3-*y*_ layer have not been discussed in detail. The interaction between Pd atoms in the perovskite host is especially important considering the possibility of nanoscale spinodal decomposition as pointed out by Kizaki et al. [12]. Therefore, we systemically studied the relative stability of the Pd_*m*_V_O*n*_-containing surfaces (*m* =1 and 2 and *n* =0, 1, and 2) in our present work to investigate possible phases appearing in steps to prepare catalysts at high temperature in air.

## Methods

### Model and computation

We have calculated the lattice constants [13] of LFO and the segregation tendency of Pd at two terminations of the perovskite surfaces with and without V_O_ by using state [14,15] and quantum ESPRESSO (QE) [16] codes. We found that both state and QE codes yielded the similar bulk lattice constants and caused the segregation behavior of Pd, which was a strong indication that both codes could admirably describe the properties of Pd incorporated in the LaFe_1-*x*_Pd_*x*_O_3-*y*_ surfaces. Here, we employed the state code to do the first-principles calculations. The ion-electron interactions were described using ultrasoft pseudopotentials [17], and the exchange and correlation potential was represented by a generalized gradient approximation (GGA) in the Perdew-Burke-Ernzerhof formula [18]. DFT calculations with Hubbard correction (DFT+U) are known to correct the bandgap and magnetic moment in local-density approximation and generalized gradient approximation calculations. This method can yield reasonable agreement with the experimental results. We omitted DFT+U from this work because Hamada et al. verified [10] that electronic structures with DFT+U are qualitatively the same as those in GGA calculations, and they have not changed their conclusions. However, since the relative energies that are used to determine the stability of perovskite surfaces might be influenced by the exchange and correlation potential, even though DFT+U fails to give better results than GGA calculations to predict the phase stability of hematite surfaces [19], we still intend to investigate the effect of DFT+U in later work. The original unit cell used to construct the LFO perovskite surface was a GdFeO_3_-type orthorhombic unit cell (adapted from Figure one in [13]), in which the local magnetic moments of Fe are aligned in G-type anti-ferromagnetic order. The relaxed lattice constants for *a*, *b*, and *c* in bulk LFO correspond to 0.575, 0.559, and 0.792 nm, respectively, which are in reasonable agreement with the experimental values [20] of 0.558, 0.556, and 0.785 nm. The cutoff energies for the wave function and augmentation charge density are 25 Ry for the former and 225 Ry for the latter.

**Figure 1:**
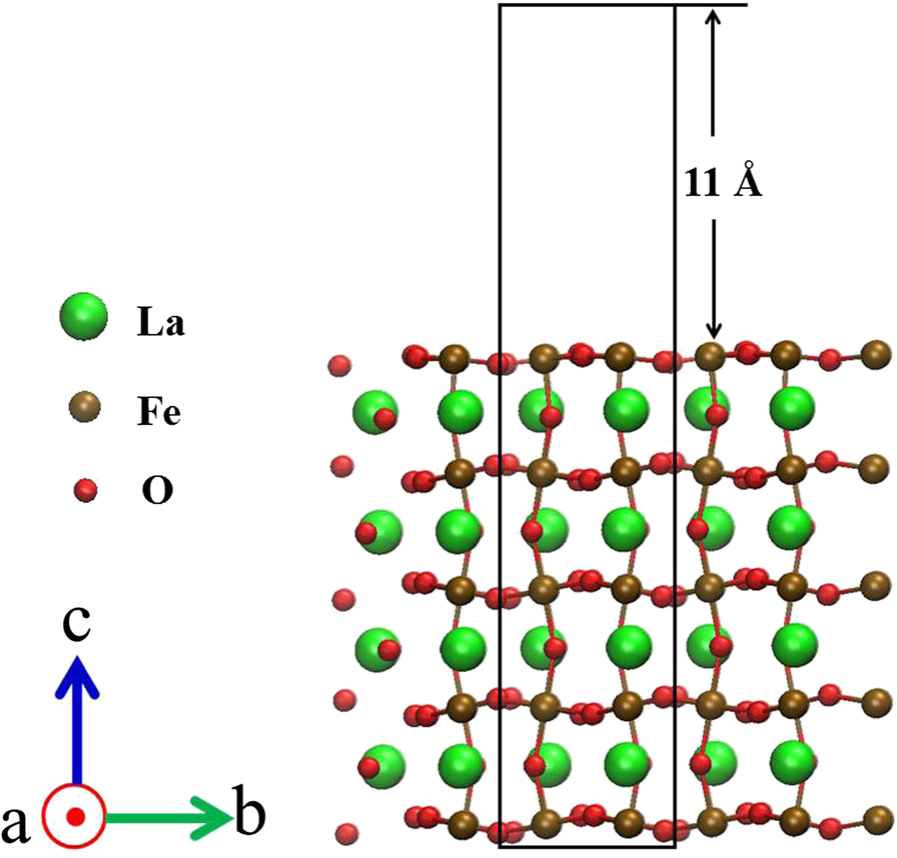
**Side views of FeO**_**2**_**-terminated surfaces.** A vacuum region with a thickness of 11 Å is placed above the top surface. The green, brown, and red spheres correspond to La, Fe, and O, respectively. The computational unit cell used in the present research is indicated by the black solid line. The bottom two layers were fixed during optimization.

We modeled the $\sqrt{2}\times \sqrt{2}$ LFO (001) surface by using a repeated slab model. Hamada et al. [10] had already shown and we confirmed [13] that one V_O_ formed in the LFO (001) surface promoted the tendency of Pd to segregate in bulk. Moreover, we further demonstrated that Pd has the strongest tendency to segregate at FeO_2_-terminated surfaces containing V_O_s, in comparison with three other surfaces, i.e., LaO-terminated surfaces with and without V_O_s and the perfect FeO_2_-terminated surfaces. Additionally, Lee et al. [21] calculated a surface phase diagram of the LFO (010) surface and argued that the LaO-terminated surface could be predicted to be stable at lower temperature (773 K), which was in agreement with the previous experimental results measured by X-ray photoelectron spectra [22,23]. In contrast, the FeO_2_-terminated surface became dominant at high temperatures (>1,500 K). Therefore, thermal treatment at high temperature is essential to make FeO_2_-terminated surfaces more stable. We thus examined FeO_2_-terminated surfaces in this work. The atomic configuration for a pristine FeO_2_-terminated surface is in Figure 1, which was obtained with visual molecular dynamics [24]. Our repeated slab model consisted of nine atomic layers, i.e., five FeO_2_ layers and four LaO layers. Further, one unit cell contained eight La atoms, 10 Fe atoms, and 28 O atoms in total. Brillouin-zone integration was carried out within a Monkhorst-Pack [25] scheme using a uniform (4 × 4 × 1) mesh. We inserted a vacuum region of 11 Å to minimize the interaction between two adjacent slabs. We fixed the two bottom layers to the bulk coordinates during the geometry optimizations and allowed atomic relaxation for the rest of the layers.

## Results and discussion

**Figure 2:**
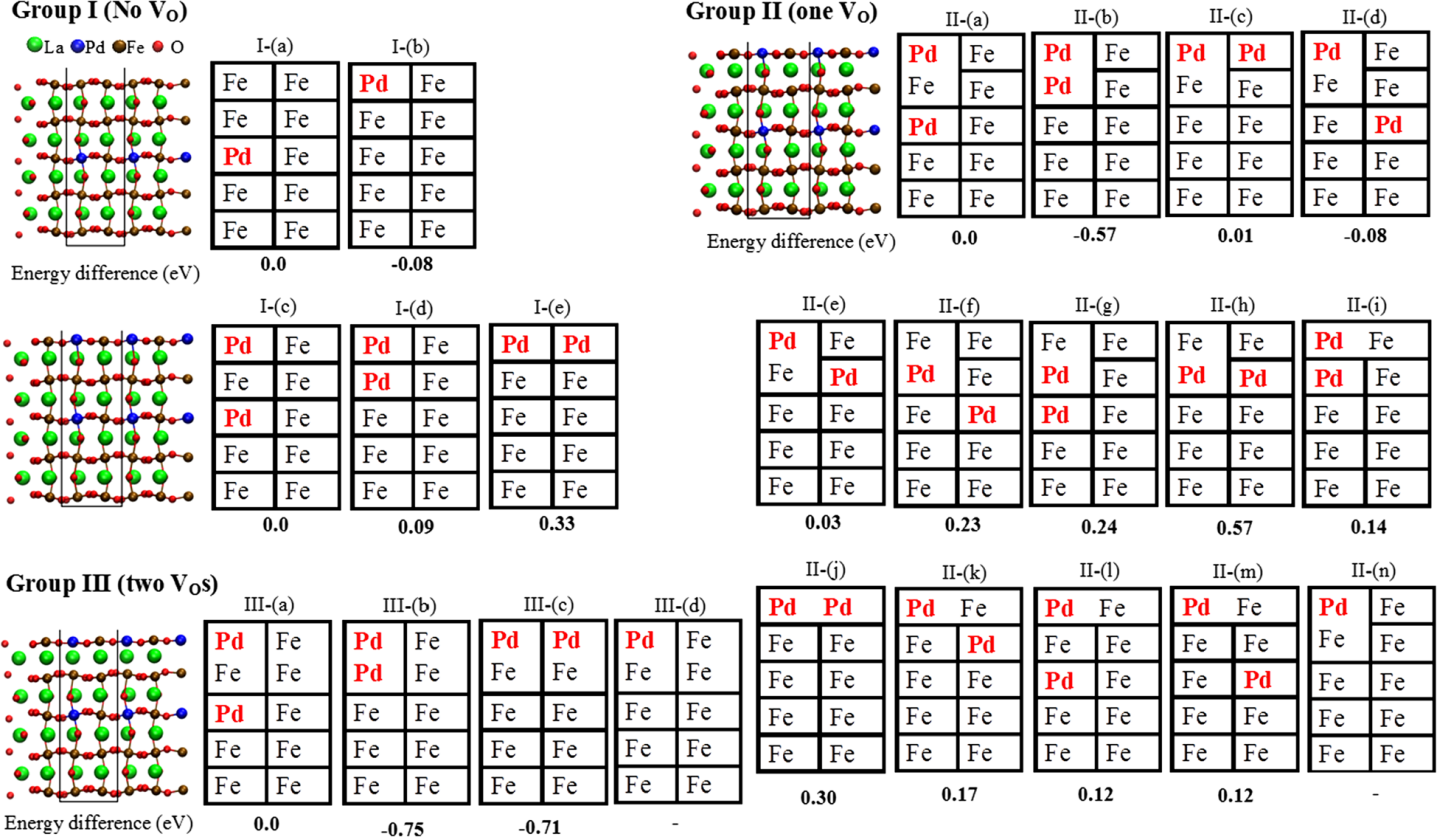
**Simplified 2D tables that represent complicated structures of perovskite surfaces containing Pd**_***n ***_**(*n*=1 and 2).** Groups I to III are for the geometries with no V_O_, one V_O_, and two V_O_s, respectively. The atomic configurations for each group, which are schematically represented by the table of panel (**a**), are indicated by the ball and stick model. The uncapping unit cell is indicated by the black line as seen in Figure 1. The rows containing Fe (Pd) in each table represent FeO_2_ (PdO_2_) layers, and the vertical lines represent O atoms in FeO_2_ (PdO_2_) layers. The horizontal lines represent O atoms in LaO layers (La atoms are not explicitly shown). The absence of vertical (horizontal) lines means V_O_ forming at the surface (subsurface) site. The calculated difference in energy (in eV) for each panel relative to the total energy of the surface in panel (a) is also listed.

**Figure 3:**
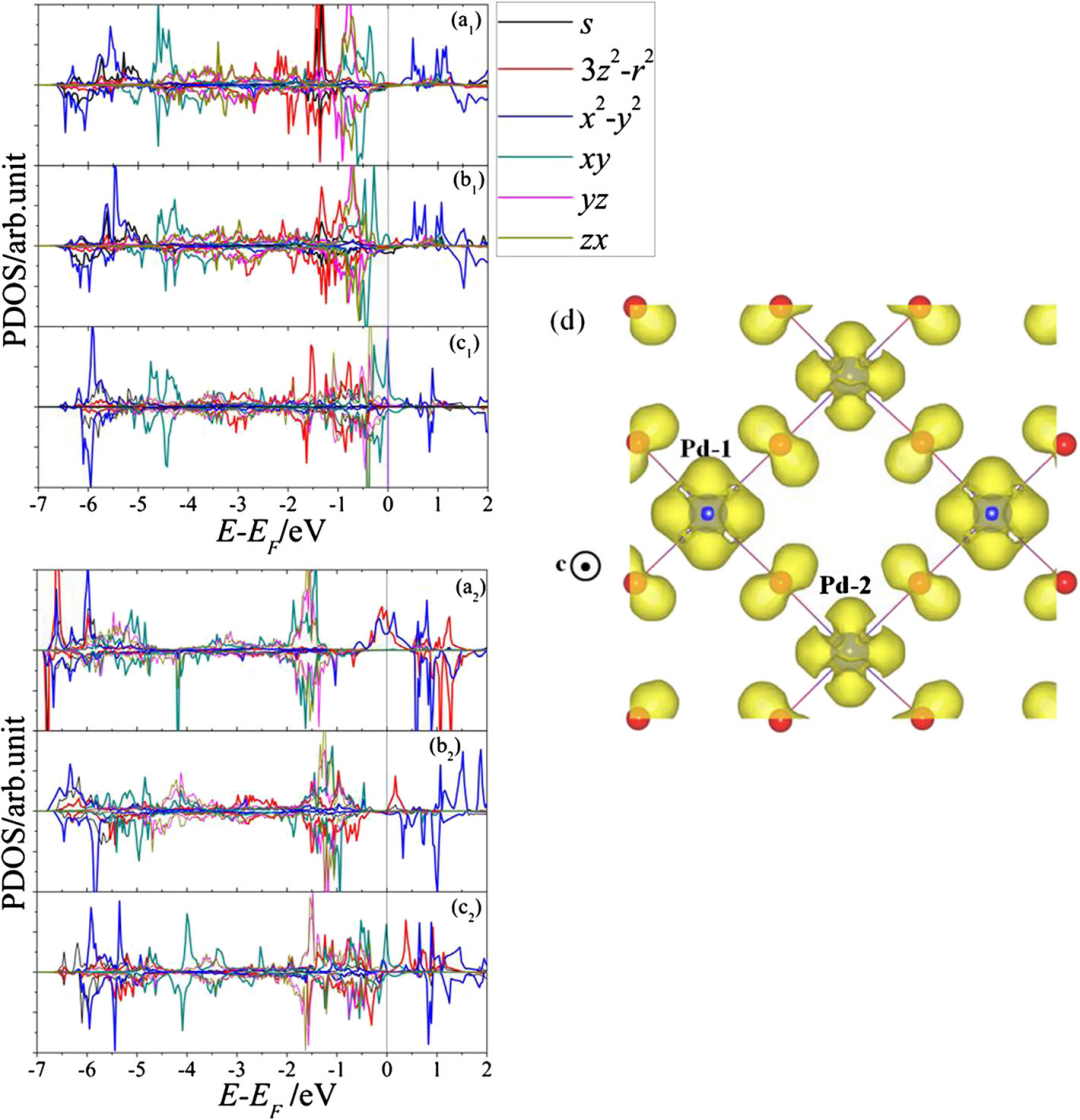
**Calculated projected density of states (PDOS) of two Pd atoms.** Panels (a_1_) to (c_1_) are the PDOSs for Pd-1 located at the top-left site of Figure 2 group II (**a**) to (**c**). Panels (a_2_) to (c_2_) represent the PDOSs of Pd-2, which is located at the third FeO_2_ layer (a_2_), at the subsurface (b_2_), or the first FeO_2_ layer (c_2_). Positive (negative) values refer to spin-up (spin-down) states. The line through the zero point on the horizontal axis represents the Fermi level. (**d**) is the top view of the calculated isosurface of the partial charge density of the PdO_2_-terminated surface containing V_O_ in the spin-up state in an energy window of (-0.1, 0.1) eV. The yellow-curved isosurfaces stand for the charge density of 0.6 a.u.^-3^.

Simplified 2D tables that represent the complicated atomic configurations of perovskite surfaces have been provided in Figure 2 to clarify the discussion. Configurations with negative formation energies are more stable than the reference configuration. One Pd segregating from the third FeO_2_ layer to the surface just releases an energy of about 0.08 eV [13] (Figure 2 group I (a) and (b)) as we demonstrated without V_O_s. However, when one Pd has already segregated on the topmost site of a perfect LFO surface, the additional Pd prefers to stay inside the bulk rather than segregate onto the surface as shown in Figures 2 group I (c) to (e). One first has to determine the positions of V_O_s and Pd atoms in studying the effect induced by V_O_s on the stability of Pd atoms. We have to calculate all the possible configurations containing V_O_s and Pd. Hamada et al. [10] pointed out that the most stable site for V_O_s is the topmost surface for pristine LFO and the subsurface (LaO layer) O site for Pd located in the first layer of the LFO surface. We considered V_O_s formed at those two possible sites along with various configurations of Pd atoms at the FeO_2_-terminated surface. We set the first configuration in panel (a) in group II to the reference state in which one Pd atom was located in the first FeO_2_ layer, the second Pd atom was in the third FeO_2_ layer, and a V_O_ was located in the first LaO layer just under the first Pd. The positions of the first Pd atom and V_O_ were found to have the most stable configuration. Positive formation energies for panels (i) to (m) in group II indicate that V_O_s that formed on the topmost surface is unstable. However, the most stable state was found with a formation energy of about -0.57 eV when a V_O_ was located at the subsurface nearly at the center of two Pd atoms, as seen in Figure 2 group II (b). However, one of the Pd atoms tended to be buried in the second FeO_2_ layer (panel (b)) rather than exposed to the vacuum (panel (c) in group II), and the energy discrepancy between panels (b) and (c) was as large as 0.58 eV. We analyzed the projected density of state (PDOS) of the two Pd atoms in the V_O_-containing surfaces to understand the origin for the difference in stability between panels group II (b) and (c). All the results are presented in Figure 3. We denoted the Pd located at the top-left site in the unit cell in Figure 2 group II (a) to (c) as Pd-1 and the other one as Pd-2. Where Pd-2 stayed inside the bulk (Figure 2 group II (a)), the PDOS of Pd-1 looked similar to that in Figure five (e) in [13], i.e., a single Pd at the first FeO_2_ layer with one V_O_ beneath it. The V_O_ beneath Pd-1 reduces hybridization between the Pd *d*_3*z*_^2^_-*r*_^2^ state and O *p* state, leading to significant stabilization of the *d*_3*z*_^2^_-*r*_^2^ state. The degenerated *e*_*g*_ states of 4*d*-orbitals for Pd-2 are singly occupied (Figure 3a_2_). When Pd-2 replaces the Fe atom at the second FeO_2_ layer (Figure 2b), the change in PDOS of Pd-1 seems rather small and similar to that in panel (a_1_). However, the degeneracy of the *e*_*g*_ state is lifted for Pd-2 because of the missing apical oxygen atom, leading to a downward shift in *d*_3*z*_^2^_-*r*_^2^ beneath the Fermi level, except for a small antibonding state near the Fermi level associated with hybridization between the Pd *d*_3*z*_^2^_-*r*_^2^ and *p* state of oxygen atom beneath it. The *t*_2*g*_ states are also fully occupied in the form of a stable closed shell. The degeneracy of the *e*_*g*_ state is lifted due to the lowering of symmetry at the surface for Pd-2 located at the first FeO_2_ layer (Figure 2 group II (c)). However, as there is another O at the subsurface, a much stronger antibonding Pd *d*_3*z*_^2^_-*r*_^2^ state appears near the Fermi level in contrast to that in panel (b_2_). Additionally, the *d*_*xy*_ state remarkably increases in energy due to increased hybridization between the Pd-*d*_*xy*_ and O-*p*_*y*/*x*_ states, and an especially sharp peak emerges at the Fermi level in the spin-up state. The Pd *d*_*xy*_ state also appears near the Fermi level for Pd-1 as shown in panel (c_1_). The corresponding partial charge density for the peak at the Fermi level has been drawn on the (001) plane in panel (d). The spin-up partial charge density exhibits strong antibonding states in the form of pdπ* bonds between Pd and O in the energy window from -0.1 to +0.1 eV relative to the Fermi energy. As a result, the additional Pd at the neighboring surface site is less stable than that at the second FeO_2_ layer.

Since V_O_s are more likely to form at the subsurface (LaO layer) than the surface in the Pd-containing FeO_2_-terminated surface, we placed another V_O_ in the same LaO layer (Figure 2 group III (a) to (c)). If two V_O_s are both located at the subsurface, the second Pd atom tends to substitute the Fe atom either at the second FeO_2_ layer forming a pair of Pd atoms (Figure 2 group III (b)) or on the surface forming the PdO_2_ layer (Figure 2 group III (c)). The difference in energy between these two configurations is less than 0.05 eV. Thus, the additional V_O_ stabilizes the PdO_2_-layer exposed to the vacuum.

**Figure 4:**
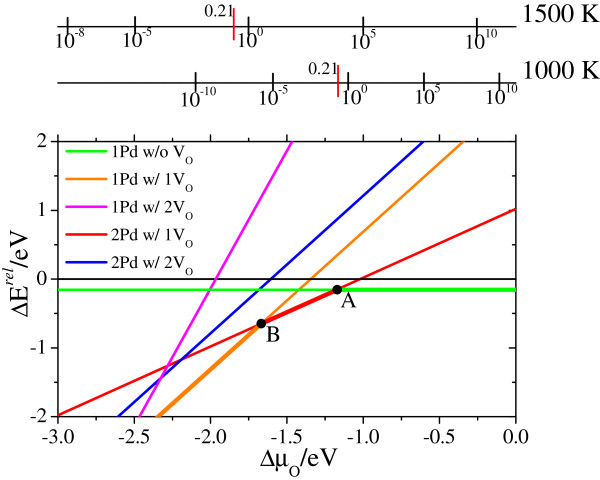
**Calculated relative energies of five LFO surfaces containing Pd**_***m***_**V**_**O*n***_**.** This is with respect to the dissolution phase of the LaFe_1-*x*_Pd_*x*_O_3_ slab as a function of Δ*μ*_*O*_ and oxygen partial pressure at high temperatures.

Thus far, we have assumed the existence of V_O_. However, the concentration of V_O_s depends on their formation energy. Therefore, we have to verify the stability of surfaces containing V_O_(s) with different concentrations of Pd by taking the formation energy of V_O_s into account to further strengthen our conclusion. We calculated the relative energies of surfaces containing Pd_*m*_V_O*n*_ (*m* =1 and 2 and *n* =0, 1, and 2) relative to the perfect slab (without V_O_s) with Pd inside the bulk of LFO (see Figure 2 group I (a)). Note that the systems we have discussed here are surfaces with Pd atom(s) and V_O_(s) located on/near the surface. The relative energies (*ΔE*^rel^) as a function of the chemical potential of oxygen (Δ*μ*_*O*_) are shown in Figure 4. The corresponding geometries for the Pd_*m*_V_O*n*_-containing surfaces are all included in Figure 2. Since two Pd atoms fail to segregate near/at the surface adjacently without V_O_s, the results for the Pd_2_-containing perfect surface excluded from Figure 4. The *ΔE*^rel^ for each colored line is calculated as:

(1) $$\begin{array}{l} \mathrm{Green}\;\mathrm{line}:\Delta E^{\mathrm{rel}}\\ \;=2E_{{\mathrm{PdFeO}}_{4}/{\left(\mathrm{LaO}\right)}_{2}/{\left({\mathrm{FeO}}_{2}\right)}_{2}/{\left({\mathrm{LaFeO}}_{3}\right)}_{6}}^{\mathrm{tot}}-2E_{\mathrm{ref}}^{\mathrm{tot}}, \end{array}$$

(2) $$\begin{array}{l} \mathrm{Green}\;\mathrm{line}:\Delta E^{\mathrm{rel}}\\ \;=2E_{{\mathrm{PdFeO}}_{4}/{\mathrm{La}}_{2}O_{1}/{\left({\mathrm{FeO}}_{2}\right)}_{2}/{\left({\mathrm{LaFeO}}_{3}\right)}_{6}}^{\mathrm{tot}}-2E_{\mathrm{ref}}^{\mathrm{tot}}\\ \;+2\mu_{O}, \end{array}$$

(3) $$\begin{array}{l} \mathrm{Pink}\;\mathrm{line}:\Delta E^{\mathrm{rel}}\\ \;=2E_{{\mathrm{PdFeO}}_{4}/{\mathrm{La}}_{2}/{\left({\mathrm{FeO}}_{2}\right)}_{2}/{\left({\mathrm{LaFeO}}_{3}\right)}_{6}}^{\mathrm{tot}}-2E_{\mathrm{ref}}^{\mathrm{tot}}\\ \;+4\mu_{O}, \end{array}$$

(4) $$\begin{array}{l} \mathrm{Red}\;\mathrm{line}:\Delta E^{\mathrm{rel}}\\ \;=E_{{\mathrm{PdFeO}}_{4}/{\mathrm{La}}_{2}O_{1}/{\mathrm{PdFeO}}_{4}/{\left({\mathrm{LaFeO}}_{3}\right)}_{6}}^{\mathrm{tot}}\\ \;+E_{{\left({\mathrm{FeO}}_{2}\right)}_{2}/{\left({\mathrm{LaFeO}}_{3}\right)}_{8}}^{\mathrm{tot}}-2E_{\mathrm{ref}}^{\mathrm{tot}}+\mu_{O}, \end{array}$$

(5) $$\begin{array}{l} \mathrm{Blue}\;\mathrm{line}:\Delta E^{\mathrm{rel}}\\ \;=E_{{\mathrm{PdFeO}}_{4}/{\mathrm{La}}_{2}/{\mathrm{PdFeO}}_{4}/{\left({\mathrm{LaFeO}}_{3}\right)}_{6}}^{\mathrm{tot}}\\ \;+E_{{\left({\mathrm{FeO}}_{2}\right)}_{2}/{\left({\mathrm{LaFeO}}_{3}\right)}_{8}}^{\mathrm{tot}}-2E_{\mathrm{ref}}^{\mathrm{tot}}+2\mu_{O}, \end{array}$$

where the first items in Equations 1 to 5 on the right-hand side are the total energies of the Pd_*m*_V_O*n*_-containing (*m* =1 and 2 and *n* =0, 1, and 2) surfaces, with their subscripts describing their compositions. $E_{\mathrm{ref}}^{\mathrm{tot}}$ represents the total energy of the reference surface that contains one solid-solution state of Pd inside the bulk. The $E_{{\left({\mathrm{FeO}}_{2}\right)}_{2}/{\left({\mathrm{LaFeO}}_{3}\right)}_{8}}^{\mathrm{tot}}$ in Equations 4 and 5 is the total energy of the pristine FeO_2_-terminated surface. The *μ*_O_ is the chemical potential of oxygen. The chemical potentials of oxygen in the LFO bulk and gas phase are equal under equilibrium conditions. The *μ*_O_ based on an ideal gas approximation is directly connected with the partial pressure (*p* (O_2_)) and temperature (*T*) by

(6) $$\mu_{O_{2}}^{\mathrm{gas}}\left(T,p\right)=\mu_{O_{2}}^{\mathrm{gas}}\left(T,p^{0}\right)+\mathrm{kT}\ln\left(\frac{p}{p^{o}}\right),$$

(7) $$\begin{array}{l} \Delta \mu_{O}^{\mathrm{gas}}\left(T,p\right)=\frac{1}{2}\left(\mu_{O_{2}}^{\mathrm{gas}}\left(T,p\right)-E_{O_{2}}^{\mathrm{gas}}\right)\\ \;=\Delta \mu_{O}^{\mathrm{gas}}\left(T,p^{0}\right)+\frac{1}{2}\mathrm{kT}\ln\left(\frac{p}{p^{o}}\right), \end{array}$$

in which *k* is the Boltzmann constant and *p*^0^ is taken to be the standard pressure. $\mu_{O_{2}}^{\mathrm{gas}}$ is the total energy of an isolated O_2_ molecule. The $\Delta \mu_{O}^{\mathrm{gas}}\left(T,p^{0}\right)$ item is determined by using thermodynamic data from the thermochemical tables [26]. Hence, we can obtain the formation energy of V_O_(s) based on Equations 2 to 5 by subtracting the item in Equation 1. As we assumed that the chemical potential of La would be very low in the present research, no metallic La or La oxides could precipitate on the surface although the La-O bonds were broken due to the formation of V_O_s in the first LaO layer (Figures 2 groups II and III). According to the equations, the positive *ΔE*^rel^ means the reference surface is more stable.

We can find from Figure 4 that when Δ*μ*_O_ is greater than -1.17 eV (point A), no V_O_s form on the surface. The Pd-segregated surface (Figure 2 group I (b)) is slightly more stable than the surface with Pd inside the bulk of the perovskite (Figure 2 group I (a)). This indicates that Pd preferentially stays at the first layer of the LFO surface than the bulk position to some extent. One V_O_ in the surface appears at the subsurface (LaO layer) when Δ*μ*_O_ is lower than -1.17 eV. The surface containing Pd_2_V_O_ is predicted to be stable between points A and B, indicating conditions with standard pressure at temperatures between 1,000 and 1,500 K. Two Pd atoms attract each other in such a surface by sharing one V_O_ in the first LaO layer (Figure 2 group II (b)). The Pd_1_V_O1_-containing surface (Figure 2 group II (n)) becomes dominant at Δ*μ*_O_ below -1.67 eV (point B) under standard pressure at temperatures over 1,500 K. Two V_O_s-containing surfaces are predicted to be dramatically unstable compared with the other three surfaces due to the greater formation energy of two V_O_s under the conditions given in Figure 4. The Pd_1_V_O2_-containing surface (Figure 2 group III (d)) will appear under standard pressure at temperatures far above 1,500 K (the pink line: the critical point is beyond the scale of Figure 4). The surface containing Pd_2_V_O2_ (Figure 2 group III (b)) for the blue line is predicted to be unstable under any conditions as presented in Figure 4. From what we have mentioned above, one V_O_ can be produced at the first LaO layer of the FeO_2_-terminated surfaces with segregated Pd_*m*_ (*m* =1 and 2) under reasonable working conditions, and such surfaces are predicted to be dominantly stable over a wide range of Δ*μ*_O_.

## Conclusions

We investigated what effect oxygen vacancies had on the tendency of additional Pd atoms to segregate at the LaFe_1-*x*_Pd_*x*_O_3-y_ surface, as well as compared the relative stability of FeO_2_-terminated surfaces that contained Pd_*m*_V_O*n*_ versus the oxygen chemical potential, by using first-principles theoretical calculations. We pointed out that Pd atoms repulse one another without V_O_s. However, if there are V_O_s at the subsurface layer, Pd atoms become attractive, forming a pair of Pd atoms while sharing one V_O_. Furthermore, we clarified that the FeO_2_-terminated surface containing Pd_*m*_V_O_ could be predicted to become stable over a wide range of oxygen chemical potentials below -1.17 eV. Therefore, the present results provide support that the calcination of precious metals containing catalysts at 1,073 K or high temperatures in air during the catalyst preparation step leads to the formation of oxygen vacancies near the surface and then enhances the formation of a LaPdO_3-*y*_ layer in the vicinity of the LaFeO_3_ oxide surface.

## Competing interests

The authors declare that they have no competing interests.

## Authors’ contributions

ZT, AU, IH, and SY carried out calculations with the help of HK and KI and drafted the manuscript. YM participated in the design of the study and helped to draft the manuscript. All authors read and approved the final manuscript.
